# Supplementary material for: Dust or disease? Perceptions of influenza in rural Southern Malawi
Source: PLoS One. 2019 Apr 22;14(4):e0208155. doi: 10.1371/journal.pone.0208155 (PMC6476467; doi:10.1371/journal.pone.0208155)
Supplement: S1 File — (DOCX) [file pone.0208155.s001.docx]

| Critical incident interviews Description of treatment seeking pathway – process through which research participant came to bring child to hospital. Including:  Identification of illness:   - what parent/carer thought was wrong and why - how the illness was recognised   Treatment decisions:   - when was it decided to seek treatment and why - who was consulted (e.g. relatives, community members) - use of home treatments - help sought from other providers (e.g. traditional healers) and why/why not - decision to attend the health centre - factors affecting decisions e.g. advice from family members, perceived quality of different service providers, past experience of seeking care, perceived severity of illness.   Wider treatment seeking experiences:   - Where treatment is sought and why - Past experiences with different providers | Focus group discussions **Community leaders and parents/carers**  Perceptions of influenza and treatment responses:   - Priority within the community and in relation to other illnesses - Perceived risk and severity - Prevention and treatment options - Community approaches to treatment seeking   Vaccinations:   - History of vaccination in the community (e.g. previous campaigns and attitudes towards these) - Perceived purpose of vaccinations - Common perceptions of vaccinations including reluctance or concerns - Variations in perceptions between vaccinations and groups within the community - Likely acceptability of a non-routine vaccine for influenza and factors affecting this - Probe on issues around Convenience, Confidence and Complacency   Experience of other health interventions:   - Examples of recent health interventions and experience/perceptions of these   **Traditional healers**  Perceptions of influenza and treatment responses:   - Causes and symptoms - Severity and risk - Prevention and treatment options - Community approaches to treatment seeking     Vaccinations:   - Past experience of vaccination campaigns within the district - Past experience of other health campaigns within the district - Perceptions of vaccinations – purpose, value, risks - Likely acceptability of a non-routine vaccine for influenza and factors affecting this   **Health care workers**  Perceptions of influenza:   - Prevalence of influenza and groups most at risk - Severity and prioritisation in relation to other illnesses - Prevention and treatment options - Community approaches to influenza treatment seeking   Vaccination   - Past experience of vaccination campaigns within the district and factors affecting community engagement - Past experience of other health campaigns within the district and factors affecting community engagement - Perceptions and uptake of vaccinations among parents/communities and factors affecting this - Likely acceptability of a non-routine vaccine for influenza and factors affecting this - Stigma for high risk groups | Interviews with potential FLUVAC trial participants *These interviews also asked about experiences of participation in an influenza vaccine trial; the section with these questions is not relevant to the article so is not included here.*  *Trial involvement:*   - *Process of FLUVAC trial recruitment* - *Perceived purpose of FLUVAC trial* - *Reasons for decision to be involved / to withdraw / not to participate in FLUVAC* - *People consulted/involved in decisions on FLUVAC participation* - *Perceived benefits and risks of involvement in FLUVAC* - *Past experience in trials or other research* - *Past experience and perceptions of drug or vaccine side effects* - *Communication and contact with FLUVAC trial study team*   **Perceptions of influenza:**   - Causes and symptoms - Severity and risk - Prevention and treatment options   Vaccination:   - Past experience with vaccinations - Perceived purpose of vaccinations - Perceived benefits and risks of vaccinations |
| --- | --- | --- |
